# Anti-Ro52/Anti-nuclear Matrix Protein 2 Positive Clinically Amyopathic Dermatomyositis Presented Only With Interstitial Lung Disease

**DOI:** 10.7759/cureus.42118

**Published:** 2023-07-19

**Authors:** Stratos Skrimizeas, Ilias E Dimeas, George Dimeas, Argyrios Tzouvelekis, Zoe Daniil

**Affiliations:** 1 Respiratory Medicine, University Hospital Patras, Patras, GRC; 2 Respiratory Medicine, University Hospital of Larissa, Larissa, GRC

**Keywords:** post-covid-19, corticosteroids, idiopathic inflammatory myopathies, anti-ro52, nxp-2 antibody, intrestitial lung disease, polymyositis, amyopathic dermatomyositis

## Abstract

This report contemplates a unique case of clinically amyopathic dermatomyositis (CADM) that presented as interstitial lung disease. The patient was a 55-year-old woman who showed up with progressive exercise intolerance and a dry cough without muscular or dermatological clinical manifestations. Diagnostic workup and imaging revealed the presence of interstitial lung disease, and further evaluation led to a positive autoimmune panel for anti-nuclear matrix protein 2 (anti-NXP2) and anti-Ro52 antibodies, establishing the diagnosis of anti-NXP2 plus anti-Ro52 antibodies-positive amyopathic idiopathic inflammatory dermatomyositis. The patient was started on intravenous corticosteroids. She showed improvement on her chest X-ray (CXR) and was then switched to oral corticosteroids. After six months of steroid treatment, corticosteroids were stopped, and the patient was re-evaluated one month later disease relapse.

## Introduction

Dermatomyositis/polymyositis is a rare immune-mediated disorder belonging to the idiopathic inflammatory myopathies (IIMs) that present with a skin rash and weakness of the proximal limb and neck muscles. Heliotrope rash and Gottron sign/papules are the pathognomonic cutaneous manifestations [1]. Several autoantibodies, each associated with a distinct clinical phenotype, underlie the disease [2]. However, many new variants of this condition have been described, such as clinically amyopathic dermatomyositis (CADM). Patients with amyopathic dermatomyositis are devoid of clinical manifestations or laboratory evidence of muscle involvement [3].

Our report discusses a specific subtype of the disease, anti-Ro52/anti-nuclear matrix protein 2 (anti-NXP2) dermatomyositis, based on the autoantibody profile of the patient [4,5], with distinct clinical implications, as improvement was shown without a second immunosuppressive agent or intravenous immunoglobulins. Hopefully, this article will raise awareness about patients with dermatomyositis presenting with atypical symptoms, as it is a disease that, despite its rarity, can seriously affect patients’ lives, so prompt diagnosis and management are important [6].

## Case presentation

A 55-year-old female teacher, an ex-smoker (45 pack years), was referred to the emergency department of our hospital after reporting a significant decrease in exercise tolerance starting two months ago, from running 5 km to only 1 km. Her main symptoms were mild shoulder discomfort and a dry, non-productive cough without hemoptysis or dyspnea. Additionally, she mentioned recovery from a mild COVID-19 infection that had been managed conservatively at home three months ago. Her past medical history includes thyroxine replacement therapy for nine years following a thyroidectomy. The patient was admitted to the Department of Respiratory Medicine for further evaluation.

Upon admission, the patient was afebrile and normopnoic. Psoriatic-like rashes were observed on the extensor surfaces of the elbows. The cardiac examination did not reveal any pathological findings. On auscultation, velcro-like crackles were heard over the bases of both lungs. Peripheral lymph nodes were normal. No evidence of muscular weakness was noted. The rest of the physical examination was unremarkable.

A hypoxemic respiratory failure with an elevated alveolar-arterial gradient was noted (Table 1) according to arterial blood gas analysis, necessitating oxygen supplementation. The patient was hemodynamically stable but had tachycardia (heart rate = 116 beats per minute). The complete blood cell count and biochemical panel were within the normal range except for a raised erythrocyte sedimentation rate (ESR).

**Table 1:** Admission blood laboratory exams pO_2_: partial pressure of O_2_, pCO_2_: partial pressure of CO_2_, pA-aO_2_:alveolar–arterial gradient, ESR: erythrocyte sedimentation rate.

Value	Patient’s value	Normal range
pO_2_	55 mmHg	>75 mmHg
pCO_2_	37 mmHg	35–45 mmHg
pA-aO_2_	48.5 mmHg	<17.8 mmHg
ESR	120 mm/hr	<30 mm/hr

**Figure 1:**
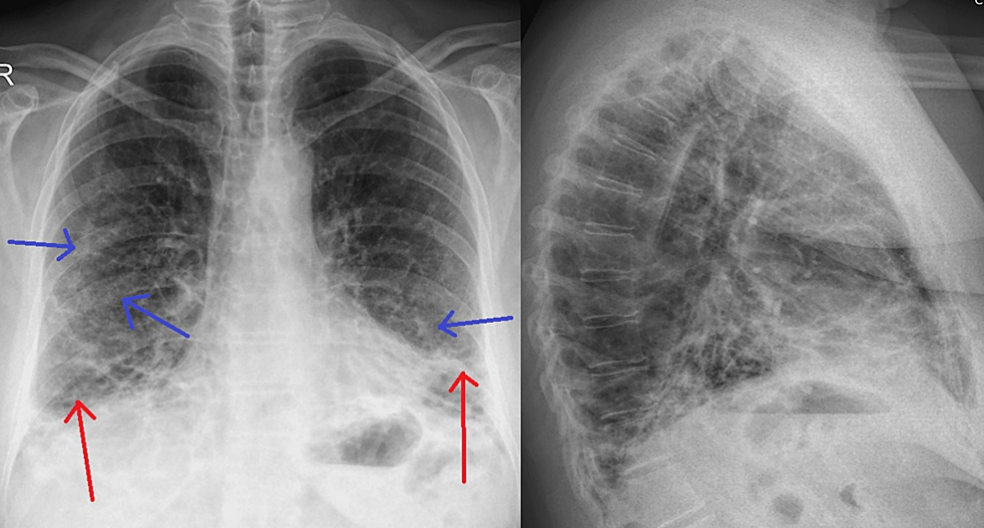
Admission chest X-ray Asymmetrical basal predominant reticular pattern (red arrows) with few areas of alveolar infiltrates (blue arrows) bilaterally.

**Figure 2:**
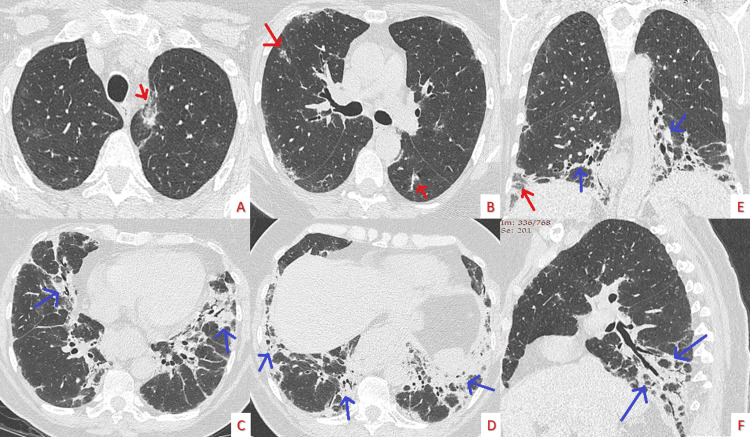
Admission chest computed tomography Bilateral ground glass opacities (red arrows) and patchy consolidations (blue arrows) with a central irregular peribronchovascular and perilobular distribution; axial slices from lung apices to bases (A-D), coronal image (E), sagittal image (F).

A chest X-ray (CXR) was performed (Figure 1), which showed an asymmetrical basal predominant reticular pattern with few areas of alveolar infiltrates bilaterally. Subsequently, a high-resolution computed tomography (HRCT; Figure 2) showed bilateral ground glass opacities and patchy consolidations with a central irregular peribronchovascular and perilobular distribution, directing to a provisional diagnosis of interstitial lung disease, possibly organizing pneumonia (OP).

Bronchoscopy demonstrated no endobronchial abnormalities. Bronchoalveolar lavage (BAL) from the right lower lobe revealed no alveolar hemorrhage and was sent for further evaluation. The polymerase chain reaction for acid-resistant bacteria and acid-fast stains was negative. Additionally, the BAL differential cell count showed lymphocytic predominance (Table 2).

**Table 2:** Bronchoalveolar lavage cell count

Cell	Percentage	Reference range
Lymphocytes	54%	5–15%
Polymorphonuclears	30%	1–5%
Monocytes	3,5%	1–3%
Eosinophils	0,5%	0–1%
Macrophages	12%	70–90%

A complete immunological panel was conducted (Tables 3-4).

**Table 3:** Autoimmune panel - anti-nuclear antibodies dsDNA: double stranded DNA, SS-A: Sjögren's syndrome A, SS-B: Sjögren's syndrome B, RNP/Sm: ribonucleoprotein/Smith, Sm: Smith, CENP: centromeric protein, PML: progressive multifocal leukoencephalopathy, Scl: scleroderma, RP: RNA polymerase.

Anti-nuclear antibody	Result	Value
dsDNA	Negative	AU: 0; cutoff<11AU
Nucleosomes	Negative	AU: 0; cutoff<11AU
Histones	Negative	AU: 1; cutoff<11AU
SS-A	Negative	AU: 2; cutoff<11AU
Ro-52	Positive	AU: 12; cutoff<11AU
SS-B	Negative	AU: 0; cutoff<11AU
RNP/Sm	Negative	AU: 1; cutoff<11AU
Sm	Negative	AU: 2; cutoff<11AU
Mi-2a	Negative	AU: 4; cutoff<11AU
Mi-2b	Negative	AU: 1; cutoff<11AU
Ku	Negative	AU: 1; cutoff<11AU
CENP A	Negative	AU: 1; cutoff<11AU
CENP B	Negative	AU: 2; cutoff<11AU
Sp100	Negative	AU: 4; cutoff<11AU
PML	Negative	AU: 1; cutoff<11AU
Scl-70	Negative	AU: 0; cutoff<11AU
PM100	Negative	AU: 2; cutoff<11AU
PM75	Negative	AU: 1; cutoff<11AU
RP11	Negative	AU: 2; cutoff<11AU
RP155	Negative	AU: 4; cutoff<11AU

**Table 4:** Autoimmune panel - autoantibodies associated with autoimmune myositis TIF: transcriptional intermediary factor, MDA5: melanoma differentiation-associated gene 5, NXP2: nuclear matrix protein 2, SAE1: small ubiquitin-like modifier 1-activating enzyme, cN-1A: cytosolic 5'-nucleotidase 1A, Ks: keratan sulfate, PM-Scl: polymyositis-scleroderma, SRP: signal recognition particle, OJ: isoleucyl-transfer RNA synthetase, EJ: aminoacyl-transfer RNA synthetase.

Autoantibodies associated with autoimmune myositis	Result	Value
Mi-2a	Negative	AU: 1; cutoff<11AU
Mi-2β	Negative	AU: 3; cutoff<11AU
TIF-1γ	Negative	AU: 0; cutoff<11AU
MDA5	Negative	AU: 2; cutoff<11AU
NXP2	Positive	AU: 77; cutoff<11AU
SAE1	Negative	AU: 1; cutoff<11AU
Ku	Negative	AU: 1; cutoff<11AU
PM-Scl 100	Negative	AU: 0; cutoff<11AU
PM-Scl 75	Negative	AU: 2; cutoff<11AU
Jo-1	Negative	AU: 3; cutoff<11AU
SRP	Negative	AU: 2; cutoff<11AU
PL-7	Negative	AU: 1; cutoff<11AU
PL-12	Negative	AU: 1; cutoff<11AU
EJ	Negative	AU: 1; cutoff<11AU
OJ	Negative	AU: 1; cutoff<11AU
Ro-52	Positive	AU: 19; cutoff<11AU
cN-1A	Negative	AU: 1; cutoff<11AU
Ha	Negative	AU: 0; cutoff<11AU
Ks	Weakly negative	AU: 8; cutoff<11AU
Zo	Negative	AU: 1; cutoff<11AU

Based on the clinical presentation and diagnostic evaluation, several possible diagnoses were considered at first: (i) organizing pneumonia-like post-COVID-19 reaction; (ii) anti-Ro52 interstitial pneumonia with autoimmune features; (iii) COVID-19-related anti-NXP2 dermatomyositis-polymyositis (DM-PM); (iv) anti-NXP2 amyopathic idiopathic inflammatory myopathy (amyopathic DM-PM) presenting as interstitial lung disease with anti-Ro52 progressive phenotype; (v) anti-Ro52/Anti-NXP2 amyopathic idiopathic inflammatory myopathy (amyopathic DM-PM) with connective tissue disease overlap presenting as interstitial lung disease.

After a multidisciplinary team meeting including rheumatologists, radiologists, and immunologists, based on the clinical features (clinically amyopathic phenotype of DM) of our patient, the specific positive immunological panel results, the prolonged time between the COVID-19 positive test and the appearance of the symptoms, the HRCT scan findings, and the negative PET-CT for malignancy or inflammatory muscle tissue, the anti-NXP2 amyopathic idiopathic inflammatory myopathy (amyopathic DM-PM) presenting as interstitial lung disease with anti-Ro52 progressive phenotype was considered the most appropriate diagnosis.

SARS-CoV-2 may cause postinfectious myositis, which may range from direct virus-induced myositis to virus-triggered autoimmunity-related myositis. Patients who have both DM and COVID-19 [7] present with various cutaneous manifestations and elevated values of creatine kinase, which our patient did not have. Whether COVID-19 contributes to the occurrence of typical DM that carries myositis-specific autoantibodies remains unclear [8]. However, all reported cases occurred within a very short timeframe after vaccination or infection, contrary to our case, which presented CADM three months after COVID-19 infection.

**Figure 3:**
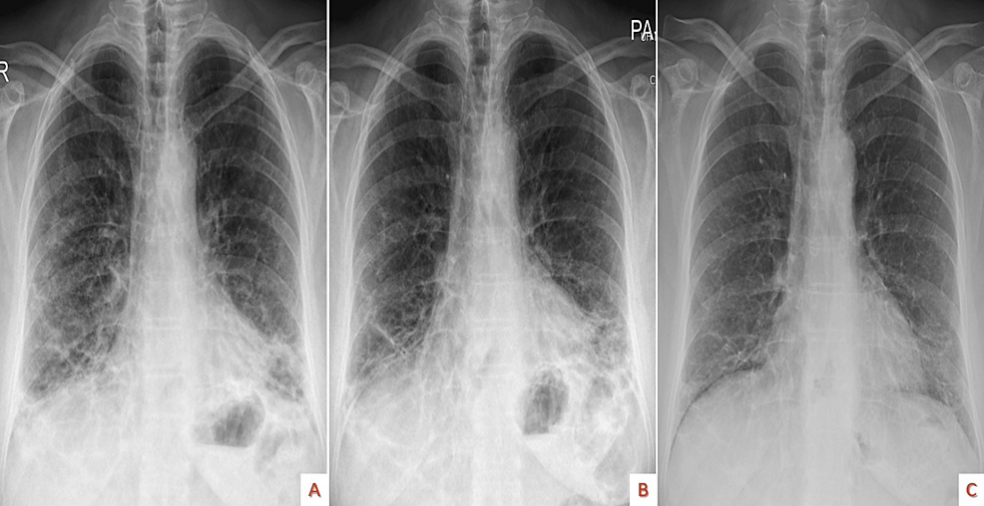
Serial inpatient follow up chest X-rays Radiological improvement with a decrease of reticulation and infiltrates, steroid naive (A), after four days of therapy (B), after eight days (C).

Even though hypoxemia indicated a severe presentation of the disease, the patient was initially only treated with steroids (iv methylprednisolone dosage almost equivalent to 1.5 mg/kg prednisolone for four days and then dosage almost equivalent to 0.5 mg/kg prednisolone for four days) with additional prophylaxis for osteoporosis and pneumocystis jirovecii pneumonia. Because of a remarkable improvement in CXR (Figure 3) and pulmonary function tests (Table 5), the patient was then switched to oral methylprednisolone.

**Table 5:** Serial improving pulmonary function tests along with decreasing corticosteroid dosage FEV_1_: forced expiratory volume in the first second, FVC: forced vital capacity, TIF: Tiffeneau-Pinelli index (FEV_1_/FVC ratio), DLCO: diffusing capacity for carbon monoxide.

Time	Methylprednisolone (mg)	FEV1 (ml)	FEV1 (%)	FVC (ml)	FVC (%)	TIF	DLCO (%)
0	0 to >125	1930	81	2260	81	85	-
4 days	125 to >32	-	-	-	-	-	-
8 days	32 to >26	2280	101	2530	95	90	-
1 months	26 to >24	2460	104	2910	104	85	77
2 months	24 to >16	2710	114	3070	110	88	76
3 months	16 to >8	2590	106	3200	111	81	81
4 months	8 to >4	2520	114	3000	115	84	78
5 months	4 ->2	-	-	-	-	-	-
6 months	2 ->0	2670	106	3090	104	86	73
7 months	0	2630	105	3120	106	84	75

**Figure 4:**
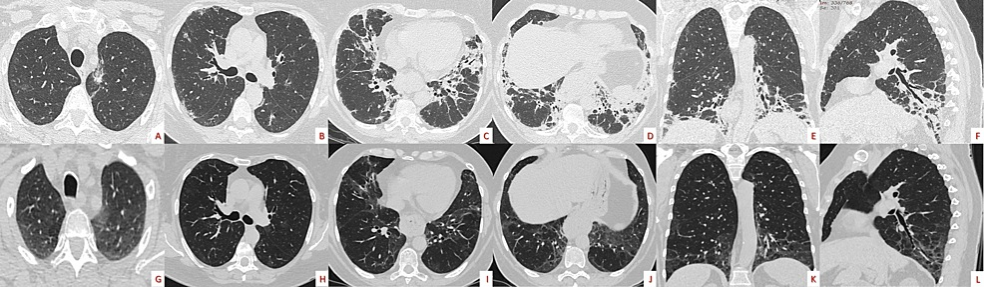
Serial follow-up high resolution computed tomographies Axial slices from lung apices to bases and coronal/sagittal images of chest computed tomographies show a radiological improvement with a decrease of bilateral ground glass opacities and patchy consolidations after one month of treatment; steroid naive (A-F), one month of treatment (G-L).

The patient was reevaluated in our outpatient clinic after one month with a marked improvement both in lung function (Table 5) and in HRCT findings (Figure 4).

**Figure 5:**
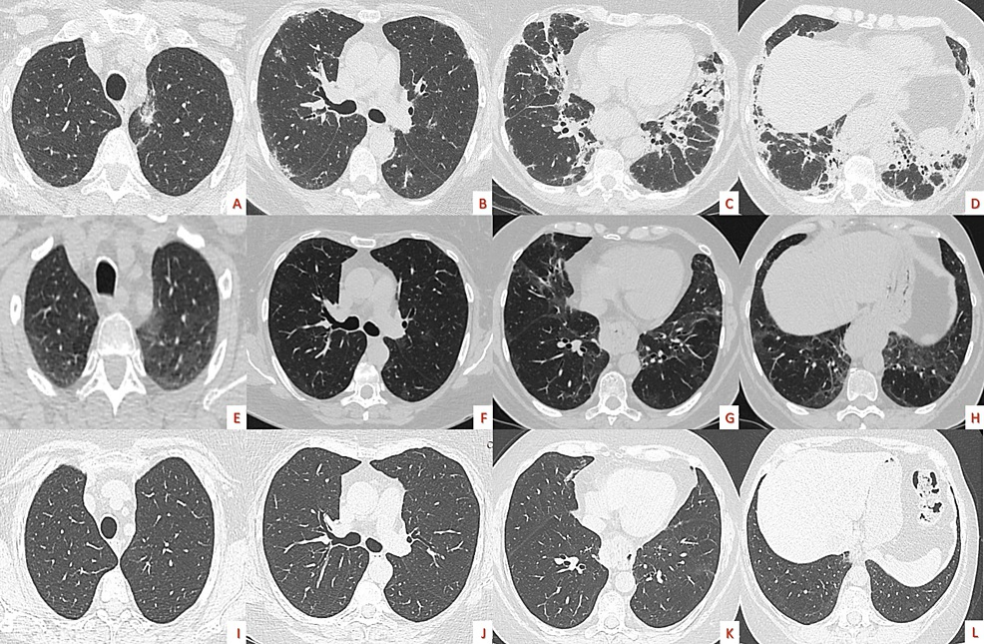
Serial follow-up high-resolution computed tomographies after therapy completion Axial slices from lung apices to bases of chest computed tomographies showing a radiological improvement; steroid naive (A-D), after one month of therapy (E-H), after four months of therapy (I-L).

**Figure 6:**
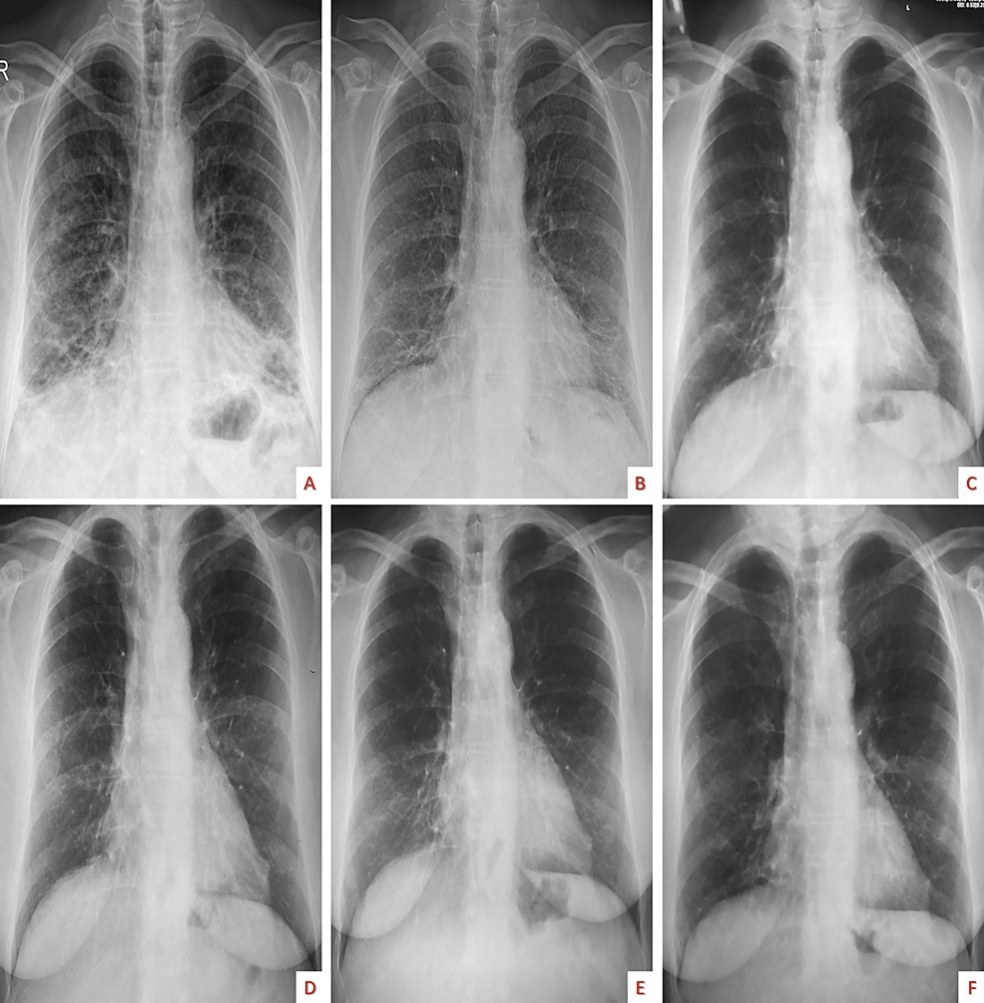
Serial chest X-rays showing a radiological improvement Steroid naive (A), after eight days of therapy (B), after two months of therapy (C), after three months of therapy (D), after six months of therapy when corticosteroids stopped (E), after seven months from admission and one-month corticosteroid free (F).

The disease remained well controlled (Figures 5-6) after six months of corticosteroids at a reduced dose without a relapse one month after corticosteroid therapy was completed.

## Discussion

Currently, anti-NXP2 dermatomyositis is a recognized subtype, as the anti-NXP2 antibody, a myositis-specific autoantibody (MSA) [2], was originally described in 1997 in a subset of patients with juvenile dermatomyositis, and the clinical manifestations included severe refractory DM with polyarthritis, joint contractures, severe calcinosis, and intestinal vasculitis [4]. The anti-NXP2 antibody is related to juvenile or adult IIMs and is associated with subcutaneous calcinosis, subcutaneous edema, and internal malignancies [9]. Anti-NXP2 recognizes the 140 kD nuclear protein NXP2, expressed in various tissues at different levels and playing an important part in diverse nuclear functions such as RNA metabolism and maintenance of nuclear architecture. Approximately 20% of juvenile DM is positive for anti-NXP2 Ab, whereas only 1.6% of adult DM is positive [10].

Anti-Ro52 antibodies belong to the anti-Ro/SSA family, a group of antibodies that has historically been used as markers of Sjögren syndrome and systemic lupus erythematosus. Ro-52 was described in 1988 as one of the proteins that Ro/SSA antibodies targeted. Ro-52 functions in cell cycle regulation, apoptotic processes, and cellular oxidative stress at the cellular level. The anti-Ro52 antibody is also part of the myositis-associated antibodies group, found in dermatomyositis. However, their low specificity in patients with non-autoimmune disorders makes anti-Ro52 diagnostic value debatable [5].

Over the past years, several researchers have tried to find associations between the anti-Ro52 antibodies and particular clinical features, mostly interstitial lung disease. Studies have shown that patients with positive anti-Ro52 antibodies have a higher frequency of rapidly progressing interstitial lung disease and mortality than those without anti-Ro52 antibodies. Hence, seropositivity for that autoantibody is of negative prognostic value.

Interstitial lung disease is a clinical entity that has been observed as one of the most frequent extramuscular manifestations of inflammatory dermatomyositis and is generally associated with poor outcomes [1]. The histopathological findings mentioned in patients with dermatomyositis-associated interstitial lung disease include usual interstitial pneumonia (UIP), OP, and, seldom, lymphocytic interstitial pneumonia (LIP) or acute interstitial pneumonia (AIP). However, nonspecific interstitial pneumonia (NSIP) is the pattern that, currently, tends to come first [11]. Radiologically, NSIP usually presents with ground-glass opacities with traction bronchiectasis, often with peribronchovascular predominance and subpleural sparing; UIP with or without honeycombing with peripheral traction bronchiectasis; and OP with peripheral consolidation with an air bronchogram and sometimes with a reversed halo sign.

It is paramount to screen interstitial lung disease (ILD) patients for dermatomyositis [12], given that timely administration of therapy improves pulmonary outcomes. The prevalence of ILD in inflammatory myopathies ranges from 19.9% to 42.6%. ILD is the first presenting clinical feature, appearing earlier than the muscular signs in 7.2% to 37.5% of cases, and ILD is more common in patients with amyopathic dermatomyositis rather than those with the classic variant of dermatomyositis.

To this end, hospitalized patients presenting with bilateral pneumonia refractory to antibiotics should be meticulously evaluated for myositis-associated ILD, as timely intervention is of vital importance [6]. It has been recently shown, in a multi-center cohort of 75 patients with myositis-associated ILD, that one-year survival was fourfold higher in the amyopathic forms of the disease compared to cases presented with myopathy [3]. The latter could be explained by a lack of awareness when systemic manifestations are occult or absent, thus leading to delayed diagnosis and therapeutic interventions.

The management of these patients depends on the disease severity at the presentation regarding pulmonary function test status, hypoxemia, and extent of CT involvement, as well as on disease progression, either with an insidious buildup or with an acute exacerbation [13]. In clinically stable cases without disease progression, a close follow-up with pulmonary function tests is suggested. When a deterioration is detected, the administration of corticosteroids is initiated with the addition of a second immunosuppressive agent. In cases with a rapidly progressive phenotype, intravenous immunoglobulins, a second steroid-sparing agent, and plasma exchange therapy should be considered. In almost all cases, the cause of death is progressive interstitial lung disease leading to respiratory failure or infections relating to immunosuppression.

## Conclusions

Dermatomyositis, a rare immune-mediated disorder manifesting with a skin rash and muscle weakness, might also present without any clinical manifestations or laboratory evidence of muscle involvement as an amyopathic variant with interstitial lung disease. A high clinical suspicion is required in order to include clinically amyopathic dermatomyositis in the differential diagnosis of progressive dyspnea in a young woman. We hope our work will help raise further awareness about the unusual presentation of autoimmune disorders through pulmonary manifestation and will be of great interest to physicians who may deal with cases like our own. Myositis-specific autoantibodies such as anti-NXP2 antibodies or myositis-associated antibodies such as anti-Ro52 play a key role in the identification of amyopathic variants, so whenever a physician is dealing with a patient with interstitial lung disease, a complete autoimmunity panel should be ordered. As for the radiological patterns in HRCT, the most prominent are peribronchovascular patchy consolidations and ground-glass opacities with irregular thickening of interlobular septa. Regarding therapeutic options, a relatively stable patient can be treated only with corticosteroids without a second immunosuppressive agent when a very close follow-up for disease progress can be secured.
